# Prohypertensive Effect of Gestational Personal Exposure to Fine Particulate Matter. Prospective Cohort Study in Non-smoking and Non-obese Pregnant Women

**DOI:** 10.1007/s12012-012-9157-z

**Published:** 2012-02-11

**Authors:** Wieslaw A. Jedrychowski, Frederica P. Perera, Umberto Maugeri, John Spengler, Elzbieta Mroz, Elzbieta Flak, Laura Stigter, Renata Majewska, Irena Kaim, Agata Sowa, Ryszard Jacek

**Affiliations:** 1 Epidemiology and Preventive Medicine, Jagiellonian University Medical College, 7, Kopernika Street, Krakòw, Poland; 2 Columbia Center for Children’s Environmental Health, Mailman School of Public Health, Columbia University Mailman School of Public Health, New York, NY USA; 3 Institute for Clinical Medicine, Research and Rehabilitation, Pavia, Italy; 4 Department of Environmental Health, Harvard School of Public Health, Boston, MA USA; 5 Obstetrics and Gynecology, Jagiellonian University Medical College, Krakòw, Poland

**Keywords:** Blood pressure, Exposure to fine particulate matter, Pregnancy, Gestational weight gain, Prepregnancy ponderal index, Environmental tobacco smoke

## Abstract

Exposure to fine particulate matter (PM) is a recognized risk factor for elevated blood pressure (BP) and cardiovascular disease in adults, and this prospective cohort study was undertaken to evaluate whether gestational exposure to PM_2.5_ has a prohypertensive effect. We measured personal exposure to fine particulate matter (PM_2.5_) by personal air monitoring in the second trimester of pregnancy among 431 women, and BP values in the third trimester were obtained from medical records of prenatal care clinics. In the general estimating equation model, the effect of PM_2.5_ on BP was adjusted for relevant covariates such as maternal age, education, parity, gestational weight gain (GWG), prepregnancy BMI, environmental tobacco smoke (ETS), and blood lead level. Systolic blood pressure (SBP) increased in a linear fashion across a dosage of PM_2.5_ and on average augmented by 6.1 mm Hg (95% CI, 0.6–11.6) with log unit of PM_2.5_ concentration. Effects of age, maternal education, prepregnancy BMI, blood lead level, and ETS were insignificant. Women with excessive gestational weight gain (>18 kg) had higher mean SBP parameters by 5.5 mmHg (95% CI, 2.7–8.3). In contrast, multiparous women had significantly lower SBP values (coeff. = −4.2 mm Hg; 95% CI, −6.8 to −1.6). Similar analysis performed for diastolic blood pressure (DBP) has demonstrated that PM_2.5_ also affected DBP parameters (coeff. = 4.1; 95% CI, −0.02 to 8.2), but at the border significance level. DBP values were positively associated with the excessive GWG (coeff. = 2.3; 95% CI, 0.3–4.4) but were inversely related to parity (coeff. = −2.7; 95% CI, −4.6 to −0.73). In the observed cohort, the exposure to fine particulate matter during pregnancy was associated with increased maternal blood pressure.

## Introduction

There has been a long and interesting debate on the potential effect of particulate matter (PM) on blood pressure (BP) and cardiovascular disorders. Of particular interest have been particles ≤10 μm in diameter (PM10) because they enter the lungs. In fact, more hazardous are fine particles (<2.5 μm; PM_2.5_) as they can penetrate deeper even in alveoli from where may be translocated into the blood stream. Many excellent reviews and meta-analyses have been published reporting statistically significant associations of cardiovascular mortality with PM exposure [1–4]. The overall evidence from the studies conducted worldwide also confirms the consistent association between increased incidence of cardiovascular events at higher PM_10_ or PM_2.5_ [5, 6].

Elevated blood pressure is an established risk factor for cardiovascular diseases and may be associated with ambient PM exposure. Several epidemiologic studies have already demonstrated that the exposure to fine particulate matter (PM_2.5_) can bring about elevated blood pressure [7–12], though some studies reported inverse relationship [13–15]. Discrepancies may result from differences in susceptibility of population groups, a relatively small number of individuals, misclassification of exposure, varying chemical composition of the PM, or lack of adjustment for relevant confounders.

It has been well documented that pregnant women are susceptible to hypertension that may require medical intervention [16]. Hypertension in pregnancy may persist and occur also at later stages of mothers’ life and their offspring. Women who are prone to gestational hypertension are older, obese/overweight, or have an excessive gestational weight gain [17, 18]. A recently published study has shown that hypertension in pregnancy may also be caused by even very low lead exposure levels [19].

The main goal of the study was to estimate the association between gestational PM_2.5_ exposure and blood pressure parameters monitored over the third trimester of pregnancy in the non-smoking and non-obese healthy pregnant women who were free from hypertension before pregnancy. The additional goal was to investigate the concentration–response relationship between the integrated personal exposure to PM_2.5_ and blood pressure to determine “safe” threshold level of PM_2.5_ for this vulnerable subset of population. The effect of exposure was adjusted for relevant confounders, such as maternal age and education, parity, prepregnancy BMI, gestational weight gain (GWG), and co-exposure to environmental tobacco smoke (ETS) and lead measured in maternal blood taken at delivery.

## Materials and methods

The present study uses data from a previously established birth cohort of children in Krakow being a part of a collaborative study with Columbia University in New York. The design of the study and the detailed selection of the population have been described in previous paper [20]. In short, pregnant women were recruited from ambulatory prenatal clinics in the first and second trimester of pregnancy. The study eligibility criteria only included women between 18 and 35 years of age, who claimed to be non-smokers, with singleton pregnancies, without illicit drug use and HIV infection, free from chronic diseases such as diabetes or hypertension, and residents of Krakow for at least 1 year prior to pregnancy. All women participating in the study had read and signed an informed consent. The study was reviewed and approved by the Bioethical Committee of the Jagiellonian University in Krakow, Poland.

Upon enrollment, a detailed questionnaire was administered to each woman to collect information on demographic data, prepregnancy weight, household characteristics, medical and reproductive history, occupational hazards, and smoking practices of others present in the home. A total of 505 pregnant women enrolled in the study; however, the present analysis was conducted in 431 women who gave birth between 37 and 43 weeks and were not obese (prepregnancy BMI <30 kg/m^2^), their non-smoker status was confirmed by blood cotinine measurement at delivery, and they had complete measurements of personal exposure to PM_2.5_.

Gestational age at birth was defined as the interval between the last day of the mother’s menstruation (LMP) and the date of birth. Maternal education level (years of schooling) was treated as a proxy for socioeconomic status. Gestational weight gain was determined as the difference between measured weight at delivery and prepregnancy weight reported by mother. Excessive GWG defined by the cutoff point of 75th percentile of gestational weight gain was equivalent to the weight gain range between 19 and 37 kg. Prepregnancy body mass index (BMI) was calculated as self-reported weight in kilograms divided by the square of height in meters.

Environmental tobacco smoke was assessed by detailed interviews on the number of cigarettes smoked daily by other household members in each pregnancy trimesters. Subsequently, an overall level of exposure to ETS was estimated from the weighted number of cigarettes smoked daily at home over the whole pregnancy period. In addition, blood cotinine level was estimated in maternal blood sample taken at delivery.

Ambulatory BP measurements in the third trimester and recorded in the medical history of prenatal clinics were performed by a medical doctor in the course of routine periodic health checkups. BP measurements were done by auscultation in the left arm and in sitting position of subjects using the standard mercury sphygmomanometer. On average, in the third trimester of pregnancy women attended about three times the prenatal care clinics (mean number of visits, 3.3; 95% CI, 3.2–3.5).

### Dosimetry of Blood Cotinine

Women at delivery provided a blood specimen, and the blood samples before laboratory analysis were stored at −70°C. The serum cotinine concentration was measured at CDC using a sensitive isotope-dilution high-performance liquid chromatographic/atmospheric pressure ionization tandem spectrometric (LC/MS/MS) procedure [21]. Limits of detection (LOD) were below 0.050 ng/mL. About 25% of specimens had cotinine levels below the LOD. Maternal blood cotinine level below 15.0 ng/L was considered the borderline separating smokers from non-smokers [22, 23].

### Dosimetry Blood Lead Level

A maternal blood sample (30–35 mL) was drawn at delivery into a vacutainer tube that had been treated with ethylene diamine tetra-acetate (EDTA). The tubes were inverted several times to mix the EDTA and the blood to prevent coagulation. Within 8 h of blood collection, the blood samples were transported to the clinical biochemistry laboratory at the University Hospital in Krakow for processing and storage. Packed red blood cells and plasma samples were separated and stored in liquid nitrogen in the laboratory prior to shipment to Columbia University. From Columbia University, portions of samples were then sent to the Center for Disease Control (CDC) for chemical analysis. Blood samples for lead analysis were refrigerated without any processing. Whole blood lead concentrations were determined using inductively coupled plasma mass spectrometry CLIA’88 method “Blood lead cadmium mercury ICPMS_ITB001A.” This multi-element analytical technique is based on quadrupole ICP-MS technology [24].

### Measurement of Prenatal Personal Exposure to Fine Particulate Matters

A Personal Environmental Monitoring Sampler (PEMS) developed by the Department of Environmental Health at Harvard University (Dr. John D Spengler) was used to measure fine particle mass. The PEMS was designed to achieve the particle target size of ≤2.5 μm at a flow rate of 2.0 L per minute (LPM). Flow rates were calibrated (with filters in place) using a bubble meter prior to the monitoring and were checked again at the change of the battery pack on the second day and at the conclusion of the 48-h monitoring period. The particles were collected on a Teflon membrane filter (37 mm Teflo™, Gelman Sciences). The combination of low pressure drop (permitting the use of a low power sampling pump), low hygroscopicity (minimizing bound water interference in mass measurements), and low trace element background (improving analytical sensitivity) of these filters make them highly appropriate for personal particle sampling.

A member of the air-monitoring staff instructed the women on how to use the personal monitor, which is a lightweight and silent device worn in a small backpack. The study participants were asked to wear the monitor during daytime hours for two consecutive days and place the monitor near their bed at night. In the statistical analysis, we included all PM_2.5_ measurements except for outliers (>99 μg/m^3^). The filters after exposure monitoring were kept in a refrigerator (temp +6°C) before mailing on dry ice to Dr. Spengler’s laboratory for gravimetric measurement of PM_2.5_.

### Statistical Methods

Descriptive statistics was calculated for all basic variables in the subgroups broken down by PM_2.5_, dichotomized at 35 μg/m^3^. Chi-square statistics and the analysis of variance tested differences in characteristics between groups. Before the main part of the statistical analysis, the nonparametric correlation and univariate analysis between the mean BP and the potential confounders were performed. In order to assess the average effect of PM exposure on BP values, the generalized estimating equations (GEE) model was used, which is applicable for repeated measurements in longitudinal studies [25].The GEE model utilizes data on all respondents, including those with incomplete records, and permits simultaneous modeling of the relationship (regression) of covariates with all BP measurements.

The model computed average effect of PM (log-transformed concentrations) measured in the second trimester of pregnancy on the BP values monitored in the third trimester and accounting for potential confounders or modifiers (maternal age and education, parity, prepregnancy body mass index, gestational weight gain (GWG), and co-exposure to ETS and lead). Due to the skewed distributions of PM and blood lead, the log-transformation of their values was used in the analysis. Covariates were introduced in the regression models as interval (age, education, BMI, blood lead, and ETS) or indicator variables (parity and GWG). Excessive GWG was defined by >75 percentile value of GWG, which corresponded to > 18 kg. Statistical analyses were performed with STATA 12.0 version for Windows.

## Results

The personal air samples collected in the second pregnancy trimester showed the geometric mean PM_2.5_ concentration of 33.6 μg/m^3^ (95% CI, 32.1–35.2 μg/m^3^). There was a significant association of and PM_2.5_ concentrations with the number of cigarettes smoked at home (Spearman correlation rho = 0.13, *p* < 0.007) and maternal blood cotinine level (rho = 0.15, *p* = 0.002). The reported number of cigarettes smoked daily at home correlated significantly with maternal blood cotinine level (rho 0.53, *p* = 0.000). In the study sample, maternal blood lead level was very low (gmean = 1.63 μg/dL; 95% CI, 1.63–1.75) and it did not correlate with PM_2.5_.

Women exposed to higher PM_2.5_ (above 35.0 μg/m^3^) had higher blood cotinine level (0.33 vs. 0.16 ng/mL, *p* = 0.083) and reported significantly greater exposure to ETS (Table 1). The mean BMI was 21.1 kg/m^2^ (95% CI, 20.8–21.1), with 26% of participants classified as overweight (BMI >25). The mean gestational weight gain (GWG) was 15.5 kg (95% CI, 14.9–15.0), and the excessive GWG gain (>18 kg) was observed in 24% of subjects.

**Table 1:** Characteristics of the study subjects grouped by exposure level to fine particulate matter PM_2.5_ in pregnancy

Variables	Total *N* = 430	PM_2.5_ (in μg/m^3)^		*p* for difference
		Lower (≤35) *N* = 235	Higher (>35) *N* = 195	
Age				
Mean	27.69	27.66	27.73	0.829
SD	3.468	3.286	3.684	
Education: years of schooling				
Mean	15.75	15.82	15.68	0.597
SD	2.728	2.581	2.899	
BMI (before pregnancy)				
Mean	21.11	21.14	21.08	0.811
SD	2.449	2.448	2.457	
Gestational weight gain				
Mean	15.51	15.29	15.76	0.322
SD	4.902	4.536	5.311	
Parity				
1 *n* (%)	272 (63.3)	151 (64.3)	121 (62.1)	0.710
2–4 *n* (%)	158 (36.7)	84 (35.7)	74 (37.9)	
Prenatal ETS^a^				
0—cigarettes *n* (%)	327 (76.0)	186 (79.1)	141 (72.3)	
≤5—cigarettes *n* (%)	74 (17.2)	39 (16.6)	35 (17.9)	0.063
>5—cigarettes *n* (%)	29 (6.7)	10 (4.3)	19 (9.7)	
Cotinine in maternal blood (ng/ml)				
≤0.146 *n* (%)	323 (75.1)	187 (79.6)	136 (69.7)	0.025
>0.146 *n* (%)	107 (24.9)	48 (20.4)	59 (30.3)	
Lead in maternal blood (μg/dL)				
≤2.100 *n* (%)	277 (76.9)	150 (77.7)	127 (76.0)	0.802
>2.100 *n* (%)	83 (23.1)	43 (22.3)	40 (24.0)	
Missing data	70	42	28	

^a^Self-reported ETS exposure

Table 2 presents arithmetic mean BP values recorded in the third trimester grouped by categorical variables. While BP values were positively associated (border significance level) with PM level (in tertiles) and the excessive GWG significantly increased with BP, parity had inverse impact. The Spearman correlation coefficients between mean BP values and interval variables are presented in Table 3. Out of the variables considered, only prepregnancy BMI had an effect only on diastolic and education on systolic BP.

**Table 2:** Mean values of systolic and diastolic BP recorded in women in the third trimester of pregnancy with 95% confidence intervals (CI) grouped by categorical variables (in brackets* p* values for significance of difference between categories)

Variables	*N*	SBB	95% CI	DBP	95% CI
PM_2.5_ (in μg/m^3)^					
<26.6	149	113.6	111.4–114.8	71.0	69.7–72.3
26.7–45.9	147	115.3	113.6–117.0	73.0	71.8–74.2
>45.9	114	116.5	114.5–118.5	72.0	70.6–73.5
		(*F* = 2.61, *p* = 0.075)		(*F* = 2.49, *p* = 0.085)	
ETS^a^					
No	311	114.7	113.5–115.8	72.3	71.4–73.1
Yes	99	116.1	113.9–118.3	71.1	69.5–72.8
		(*t* = −1.185, *p* = 0.237)		(*t* = 1.295, *p* = 0.196)	
Older siblings					
No	259	116.1	114.8–117.4	72.9	71.9–73.9
1 or more	151	113.2	111.6–114.3	70.5	69.3–71.7
		(*t* = 2.698, *p* = 0.007)		(*t* = 3.045, *p* = 0.003)	
Excessive GWG					
No	312	113.7	112.6–114.9	71.7	70.8–72.5
Yes	98	119.2	117.0–121.4	73.0	71.4–74.7
		(*t* = −4.538, *p* = 0.000)		(*t* = −1.463, *p* = 0.144)	

Missing blood measurements for 20 persons

^a^Self-reported ETS exposure

**Table 3:** Spearman nonparametric correlation coefficients (rho) between BP parameters and interval variables

Variables	Systolic blood pressure		Diastolic blood pressure	
	Rho coeff.	*p* value	Rho coeff.	*p* value
Maternal age	−0.041*	0.412	−0.020	0.685
Maternal education	−0.104	0.036	0.070	0.160
Prepregnancy BMI	−0.080	0.105	−0.102	0.039*
Blood cotinine level	0.080	0.105	0.014	0.783
Blood lead level	0.031	0.576	0.003	0.952

* Significant at *p* < 0.05

In the GEE longitudinal models, we separately estimated the relationship between PM level and all values of SBP and DBP recorded in the course of medical checkup visits in the third trimester of pregnancy accounting for potential confounders (Tables 4, 5). The estimates obtained from the model show that the average SBP values significantly increased by 6.1 mm Hg (95% CI, 0.6–11.6) with log unit of PM_2.5_ concentration. Effects of maternal age, education, prepregnancy BMI, ETS, and blood lead level were insignificant. Women with excessive weight gain (>18 kg) had higher mean SBP values by 5.5 mmHg (95% CI, 2.7–8.3) compared with those with lower GWG. In contrast, multiparous women had significantly lower SBP (coeff. = −4.2 mm Hg (95% CI, −6.8 to −1.6). Similar analysis performed for diastolic blood pressure has demonstrated that PM_2.5_ also increased DBP parameters (coeff. = 4.1; 95% CI, −0.02 to 8.2), but the effect was at border significance level. While the excessive GWG was associated with higher DBP values (coeff. = 2.3; 95% CI, 0.3–4.4), parity correlated inversely with DBP (coeff. = −2.7; 95% CI, −4.6 to −0.73).

**Table 4:** Effect of PM_2.5_ (log-transformed) exposure in pregnancy on systolic blood pressure monitored over the third trimester of pregnancy, adjusted for the potential confounders (GEE model)

Predictors	Coeff.	*z*	*p* > *z*	[95% Conf. Interval]	
Maternal age (years)	0.349	1.78	0.075	−0.035	0.733
Education^a^	−0.380	−1.60	0.111	−0.849	0.087
Excessive GWG^b^	5.483	3.88	0.000	2.716	8.249
Prepregnancy BMI	−0.404	−1.27	0.204	−1.027	0.219
Parity^c^	−4.170	−3.17	0.002	−6.747	−1.593
ETS^d^	−0.237	−1.33	0.185	−0.586	0.113
Blood lead (log-transformed)	0.773	0.19	0.850	−7.213	8.758
PM25 (log-transformed)	6.126	2.18	0.030	0.610	11.642
_cons	110.886	11.75	0.000	92.385	129.387

^a^Years of schooling

^b^0 = gestational weight gain ≤ 18 kg, 1 = gestational weight gain > 18 kg

^c^0 = no older siblings, 1 = one or more older siblings

^d^Weighted number of cigarettes smoked daily at home over pregnancy period (self-reported)

**Table 5:** Effect of PM_2.5_ (log-transformed) exposure in pregnancy on diastolic blood pressure monitored over the third trimester of pregnancy, adjusted for the potential confounders (GEE model)

Predictors	Coeff.	z	*p* > z	[95% Conf. Interval]	
Maternal age (years)	0.117	0.80	0.422	−0.168	0.403
Education^a^	0.302	1.70	0.089	−0.046	0.650
Excessive GWG^b^	2.327	2.22	0.027	0.270	4.384
Prepregnancy BMI	−0.303	−1.28	0.201	−0.766	0.161
Parity^c^	−2.651	−2.71	0.007	−4.567	−0.734
ETS^d^	−0.190	−1.43	0.152	−0.450	0.070
Blood lead (log-transformed)	1.097	0.36	0.717	−4.842	7.035
PM25 (log-transformed)	4.083	1.95	0.051	−0.019	8.185
_cons	64.521	9.19	0.000	50.763	78.280

^a^Years of schooling

^b^0 = gestational weight gain ≤ 18 kg, 1 = gestational weight gain > 18 kg

^c^0 = no older siblings, 1 = one or more older siblings

^d^Weighted number of cigarettes smoked daily at home over pregnancy period (self-reported)

**Fig. 1:**
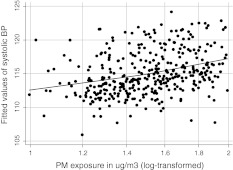
Fitted values of systolic blood pressure related to personal PM_2.5_ exposure (log-transformed values) in the third pregnancy trimester (*solid line* lowess regression line), adjusted for all covariates included in the GEE model (Table 4)

**Fig. 2:**
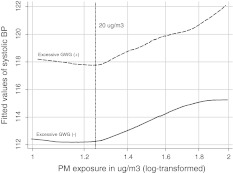
Fitted values of systolic blood pressure related to personal PM_2.5_ exposure (log-transformed values) in the third pregnancy trimester by gestational weight gain (lowess regression lines), adjusted for all covariates included in the GEE model (Table 4)

Figure 1 presents the relationship between fitted linear regression between SBP values and PM_2.5_ levels (log-transformed) adjusted for covariates in the total study sample. Instead, Fig. 2 documents the joint prohypertensive effect of PM exposure and the excessive GWG on SBP. As SBP in the third trimester of pregnancy increased linearly with logarithmic PM_2.5_ concentrations above the level of 20 μg/m^3^, this could have suggested a “safe” threshold exposure level. Contrary to subjects with low or moderate GWG, the course of the dose–effect relationship in persons with the excessive GWG did not flatten out at higher PM_2.5_ concentrations.

## Discussion

The observations from this prospective population-based cohort study of non-smoking and non-obese women free of hypertension in prepregnancy period suggest that personal daily exposure to PM_2.5_ in the second trimester of pregnancy has an effect on BP values monitored in the third trimester. There was slightly stronger relationship between PM_2.5_ and SBP than DBP, and the study suggested that women with the excessive GWG were more susceptible to prohypertensive action of particulate matter.

Blood pressure was shown to increase in a linear fashion across a logarithmically increasing personal dosage of inhaled fine-particle levels exceeding 20 μg/m^3^. While the small sample size precludes conclusions about “safe” threshold level, this value is lower than that (35 μg/m^3^) recommended by NAAQS as “safe” level [26]. The average effect of PM_2.5_ on repeated measurements of BP was estimated in the GEE longitudinal regression model and adjusted for a set of potential confounders, such as maternal age, education, parity, prepregnancy BMI, gestational weight gain, and co-exposure to lead and ETS.

The prohypertensive effect of PM in pregnancy is a very important health issue. It may increase the risk of gestational hypertension irrespective of its type. The latter may be associated with many maternal morbidities (preeclampsia, cesarean deliveries, abruptio placentae, renal dysfunction), perinatal morbidities, preterm births, and fetal growth restriction [27–29]. Moreover, in women with a history of gestational hypertension, there were observed higher rates of obesity, cardiovascular disorders, and diabetes mellitus [30].

The results of our study are consistent with the observations of other authors who show that ambient PM can adversely affect hemodynamic system in pregnancy. In the prospective study on PM_10_ and blood pressure in pregnant women, Van den Hooven et al. [31] assessed individual exposure levels of PM_10_ during pregnancy, using continuous outdoor monitoring data and considering in the GIS modeling techniques both the spatial and temporal variation in air pollution and found no association between PM_10_ exposure and the parameters of systolic and diastolic blood pressure measured in the first trimester of pregnancy. Observed 10 μg/m^3^ increase in PM_10_ levels was associated with 1.11 mm Hg (95% CI, 0.43–1.79 and 2.11 mm Hg (95% CI, 1.34–2.89) increase in systolic blood pressure in the second and third trimesters, respectively.

Our estimates of the association between air particulate matters were also consistent with the findings from a cross-sectional study of a very large population sample performed in the United States by Auchincloss et al. [32]. The authors examined 5 112 participants, 45–84 years of age, free from cardiovascular disease, where outdoor air pollution monitors were used to estimate ambient PM_2.5_ for the preceding 60 days and road traffic data to estimate local exposures to traffic-related particles. From linear regression models, it was estimated that a 10 μg/m^3^ increase in PM_2.5_ 30-day mean was associated with 0.99 mmHg higher systolic blood pressure (95% CI, −0.15 to 2.13), adjusted for age, sex, ethnicity, income, education, body mass index, diabetes, active and passive smoking, alcohol use, and physical activity. In another US population sample, Dvonch et al. [33] demonstrated also significant associations between increases in systolic BP and daily elevations in PM_2.5_ in 347 adults living in 3 distinct communities within metropolitan Detroit, Mich.

Although estimates of the association between BP and PM_2.5_ exposure in our study were based on individual personal measurement of exposure, which also accounts for indoor sources of exposure (ETS, cooking, etc.), they are to a certain extent comparable with other studies, where outdoor measurements had been done. Additional analysis performed in the subsample of our study population (comparison of 89 personal measurements with outdoor PM_2.5_ concentrations) has shown that major portion of personal PM_2.5_ variance (60%) may be explained by outdoor pollution.

In the course of our study, we did not find an association between ETS and blood pressure observed by Seki et al. [34]. The authors demonstrated that systolic morning BP measured in women at home was 4 mm Hg higher than that in the non-ETS group (116.8 vs. 113.1 mm Hg). It is possible that the effect of ETS on BP found in the latter study could have resulted from the relationship between ETS and PM_2.5_, which has not been controlled.

A recently published paper by Wells et al. [19] reported a significant association between low-level lead exposure during labor in delivery and umbilical lead levels <2 μg/dL. In our study sample, maternal blood had very low blood lead concentrations, but we found that the association between blood lead levels and blood pressure was very far from the significance level. The interpretation of the results published by Wells et al. is very difficult as blood pressure levels taken in very stressful situations during labor or at delivery do seem to produce biased BP estimates.

The biological mechanisms whereby PM_2.5_ might cause adverse health outcomes are not yet clear. PM_2.5_ is a proxy of a wide spectrum of environmental hazards that may be implicated in causing various health disorders. Fine particles are virtually always present in particle-generating processes, especially combustion processes that generate other toxic agents as well. Typically, the ambient fine-particle fraction contains constituents of tobacco and wood smoke, organic compounds, sulfates, polycyclic aromatic hydrocarbons, metals, and many others [35]. Brook and Rajagopalan [36] in their review paper discussed in a great detail three main biological pathways possibly involved in the mechanism of raised BP related to PM_2.5_ exposure. First, fine particulate matter inhaled into respiratory tract may stimulate the nasopharyngeal–pulmonary receptors of autonomic nervous system and increase vascular tone, which can lead to raised BP in a very short time [37, 38]. Second, fine particles may activate immune cells and lead to release of endogenous pro-inflammatory cytokines in lung and other tissues, which may change homeostatic responses and bring about vascular endothelial and smooth muscle dysfunction and vascular inflammation [39–41]. Third, some inhaled fine-particle constituents, such as organic compounds or metals, crossing the lung–blood barrier can directly affect vascular endothelium by inducing local oxidative stress and pro-inflammatory action [42].

At the molecular level, the oxidative stress and the consequent up-regulation of redox sensitive pathways by PM exposure seems to be the essential mechanism of pro-hypertensive reactions. The excessive production of reactive oxygen species (ROS) within the cardiovascular system causes a disturbance in the pro-oxidant–antioxidant balance and drop in the bioavailability of nitric oxide, which are important for maintaining vasomotor tone [43–45]. Mentioned earlier biological pathways of hemodynamic changes are not mutually exclusive, and setting up of particular type(s) of biological reactions is associated not only with intensity and duration of PM_2.5_ exposure, but also depends on physical and chemical composition of PM, additional co-exposure, and host characteristics.

Epigenetic studies examining gene–air pollution exposure interactions may shed more light on the biological mechanisms by which air pollution affects susceptibility. A new hypothesis suggests that a greater individual air pollution susceptibility may be linked with genomic polymorphism [46] and reduced levels of DNA methylation associated with oxidative stress. Oxidative stress from air pollution exposure can hinder the capacity of methyltransferases to interact with DNA or alter the expression of genes involved in the methylation process. In the context of potential epigenetic effects of air pollution exposure, we have to mention observations recently made by Perera et al. [47] in the New York City cohort on fetal growth and healthy development of children. In the latter study, prenatal air pollution exposure (PAHs) was significantly associated with genomic hypomethylation in cord blood white cells DNA and showed the potential for epigenetic changes.

A strength of our study is the prospective cohort design, which enabled us to measure the association between gestational personal PM_2.5_ exposure in the second trimester of pregnancy with subsequently monitored blood pressure values. A very strong advantage of the study is the application in the risk assessment of personal exposure techniques, integrating both outdoor and indoor exposures. The results were adjusted for important confounders, such as maternal age, education and prepregnancy BMI, parity, gestational weight gain, and potential co-exposure to ETS and lead. Moreover, self-reported exposure to passive smoking at home was validated by measurements of cotinine in maternal blood. Other potential confounders such as obesity, diabetes, or maternal active tobacco smoking have been removed through entry criteria. On the other side, we are aware of the limitations of our study, which are mainly related to the relatively small study sample. Although the sample of pregnant women was recruited from the general population from the Krakow inner city area inhabited by a relatively homogenous population in respect of socioeconomical variables, the sample is not representative of this population because of the entry criteria. Blood pressure measurements performed by various medical doctors from the prenatal care clinics may not precisely reflect the health outcome under question and may be biased, however, toward null. Having a longer series of BP measurements carried out by carefully standardized technical procedures would produce more precise estimates of the effect.

We believe that our study, despite the mentioned limitations, provided additional arguments for the hypothesis of the causal relationship between PM_2.5_ and its prohypertensive effects in pregnancy. The comprehensive risk assessment analysis accounting for many relevant confounders, which were seldom considered in other studies, would be helpful in preventive programs to define higher risk groups. The study results require further research on the causality of the discussed relationship since more information on health consequences of exposure to fine particulate matter combined with co-exposure to other ambient hazards or other risk factors is needed.
